# Distinct *Lotus japonicus* Transcriptomic Responses to a Spectrum of Bacteria Ranging From Symbiotic to Pathogenic

**DOI:** 10.3389/fpls.2018.01218

**Published:** 2018-08-20

**Authors:** Simon Kelly, Terry Mun, Jens Stougaard, Cécile Ben, Stig U. Andersen

**Affiliations:** ^1^Department of Molecular Biology and Genetics, Aarhus University, Aarhus, Denmark; ^2^ECOLAB, Université de Toulouse, CNRS, INP, UPS, Toulouse, France

**Keywords:** symbiosis, nodulation, RNA-seq, pathogen, legume, nitrogen fixation, plant-microbe interaction, rhizobia

## Abstract

*Lotus japonicus* is a well-studied nodulating legume and a model organism for the investigation of plant-microbe interactions. The majority of legume transcriptome studies have focused on interactions with compatible symbionts, whereas responses to non-adapted rhizobia and pathogenic bacteria have not been well-characterized. In this study, we first characterized the transcriptomic response of *L. japonicus* to its compatible symbiont, *Mesorhizobium loti* R7A, through RNA-seq analysis of various plant tissues. Early symbiotic signaling was largely Nod factor-dependent and enhanced within root hairs, and we observed large-scale transcriptional reprogramming in nodule primordia and mature nitrogen-fixing nodules. We then characterized root transcriptional responses to a spectrum of *L. japonicus* interacting bacteria ranging from semi-compatible symbionts to pathogens. *M. loti* R7A and the semi-compatible strain *Sinorhizobium fredii* HH103 showed remarkably similar responses, allowing us to identify a small number of genes potentially involved in differentiating between fully and semi-compatible symbionts. The incompatible symbiont *Bradyrhizobium elkanii* USDA61 induced a more attenuated response, but the weakest response was observed for the foliar pathogen *Pseudomonas syringae* pv. *tomato* DC3000, where the affected genes also responded to other tested bacteria, pointing to a small set of common bacterial response genes. In contrast, the root pathogen *Ralstonia solanacearum* JS763 induced a pronounced and distinct transcriptomic pathogen response, which we compared to the results of the other treatments. This comparative analysis did not support the concept that an early defense-like response is generally evoked by compatible rhizobia during establishment of symbiosis.

## Introduction

*Lotus japonicus* develops determinate root nodules in association with its compatible rhizobia *Mesorhizobium loti*. Establishment of an effective nitrogen-fixing symbiosis requires molecular communication to ensure compatibility and coordinate the developmental processes of rhizobial infection and nodule organogenesis with legume LysM-receptor kinases playing an integral role in these processes (Madsen et al., 2010; Oldroyd et al., 2011; Kelly et al., 2017a). Nod factor (NF) is the key signal molecule produced by rhizobia (Long, 1996) and is perceived by a NF receptor complex, which in *Lotus* consists of NFR1, NFR5 and SYMRK (Madsen et al., 2003; Radutoiu et al., 2003; Broghammer et al., 2012; Antolin-Llovera et al., 2014). Perception of compatible NF results in rapid physiological and transcriptional responses in the host (Desbrosses and Stougaard, 2011; Oldroyd, 2013). An additional level of compatibility scrutiny in *Lotus* occurs through the perception of rhizobial exopolysaccharides (EPSs) by the EPR3 receptor (Kelly et al., 2013; Kawaharada et al., 2015, 2017b).

Significant resources have been established to assist with investigations of *L. japonicus*. The genome sequence is available (Sato et al., 2008) as is an extensive *LORE1* retrotransposon mutant resource consisting of 150,000 lines that provides potential mutants for an estimated 90% of active protein coding genes (Malolepszy et al., 2016). A *Lotus japonicus* gene expression atlas (LjGEA) was established that incorporated and extended on available *Lotus* transcriptome data in response to rhizobia and abiotic stresses (Verdier et al., 2013). All of these genomic resources can now be accessed through *Lotus* Base (Mun et al., 2016). Comparable transcriptomic resources are available for the indeterminate nodulating model legume *Medicago truncatula* (Benedito et al., 2008) and additional significant transcriptome data for this host was provided by the specific analysis of root hairs, which revealed a role for auxin signaling in infection thread (IT) formation (Breakspear et al., 2014).

Transcriptome responses of *L. japonicus* to compatible rhizobia and arbuscular mycorrhiza form the bulk of host transcriptional data available, while responses to incompatible rhizobia and pathogens have been less well-defined. Microarray analysis has been performed on two *L. japonicus* ecotypes that show differing leaf phenotypes when challenged with *Pseudomonas syringae* pv. *tomato* DC3000 and the response to pathogenic fungal exudates has also been investigated, identifying potential defense related genes (Bordenave et al., 2013; Giovannetti et al., 2015).

The interplay between symbiotic and pathogenic responses in legumes has largely been investigated through co-inoculation experiments, which revealed a negative impact of pathogens and defense eliciting compounds on symbiotic efficiency (Lopez-Gomez et al., 2012; Chen et al., 2017). Analysis of a cDNA array of expressed sequence tags from *L. japonicus* at various time points after inoculation with *M. loti* TONO indicated that an initial defense-like transcriptional response in *L. japonicus* is subsequently dampened through symbiotic signaling. The identified defense-like genes encode hyper-sensitive related proteins, pathogenesis-related (PR) proteins, and proteins associated with phytoalexin biosynthesis and cell wall modification (Kouchi et al., 2004).

In this study, a comprehensive symbiotic transcriptomic response of *L. japonicus* to its compatible symbiont *M. loti* R7A and a spectrum of interacting bacteria was obtained through RNA-seq analysis, revealing distinct transcriptional responses and challenging the concept that an early defense-like response is evoked by compatible rhizobia in *L. japonicus* during the establishment of symbiosis.

## Materials and Methods

### Plant Material and Growth Conditions

*Lotus japonicus* ecotype Gifu (Handberg and Stougaard, 1992) was used as the wild-type plant. Seed sterilization and plant-growth setups were as previously described (Kawaharada et al., 2015). For tissue-specific analysis of transcriptome responses to *M. loti* R7A, plants were grown at 21°C with a 16 h day and 8 h night cycle. For the analysis of transcriptome responses to a spectrum of interacting bacteria, plants were grown at 25°C with a 16 h day and 8 h night cycle. Plant growth plates, each containing 10 seedlings, were inoculated with 750 μL of OD_600_ = 0.02–0.05 bacterial suspensions along the length of the root. Purified *M. loti* R7A NF was obtained as previously described (Rodpothong et al., 2009) and used at 10^-8^ M for plant inoculation.

### Bacterial Strains

Bacterial strains used in this study are listed in **Supplementary Table 1**. All strains were cultured at 28°C. *M. loti* R7A, *Bradyrhizobium elkanii* USDA61 and *Sinorhizobium fredii* HH103 were cultured in yeast mannitol broth (YMB) (Vincent, 1970), *Pseudomonas syringae* pv. *tomato* DC3000 in NYG medium (Daniels et al., 1984) and *Ralstonia solanacearum* JS763 in BGT medium (BG medium plus 0.005% tetrazolium chloride) (Saile et al., 1997). Antibiotics were added to media as required at the following concentrations: polymyxin, 50 μg mL^-1^; rifampicin, 50 μg mL^-1^; kanamycin, 50 μg mL^-1^.

### Plant Phenotyping

Phenotypic responses of *L. japonicus* to the spectrum of interacting bacteria was determined though observation of plants at weekly intervals following inoculation. Symbiotic phenotypes at 21 dpi were observed using a Zeiss Discovery V8 stereomicroscope. Disease responses, root-browning and wilting symptoms, of *L. japonicus* to *R. solanacearum* JS763 were analyzed at 5 and 29 dpi.

### Plant Tissue Harvesting

Whole root samples were harvested by removing the root tip (∼3 mm), to minimize hormone and meristematic activity specific to the root tip, and then collecting roots in tubes immersed in liquid nitrogen. Each whole root sample consisted of 10 roots. Root hairs were harvested using a previously described method (Sauviac et al., 2005). Roots were separated from shoots and root tips were removed. Ten roots were transferred to 50 mL Falcon tubes immersed in liquid nitrogen and after 1 min were twice vortexed for 15 s, tubes were returned to liquid nitrogen between vortexing. Falcon tubes were gently tapped to collect root hairs in the bottom and tweezers were used to remove roots stripped of root hairs. The process was then repeated with another 10 roots and a total of 100 roots was processed for each sample. Nodule primordia were collected at 7 dpi using a scalpel to slice through the root above and below the developing primordia. Excised primordia were collected in tubes immersed in liquid nitrogen and each sample represents the total primordia collected from 30 roots. Nodules collected at 21 dpi were gently separated from the root using tweezers and collected in tubes immersed in liquid nitrogen. Each sample represents the total nodules collected from 30 roots.

### RNA Isolation, Library Preparation, and Sequencing

Total RNA was isolated from whole root, nodule primordia, and nodule tissue samples using a NucleoSpin^®^ RNA Plant kit (Macherey-Nagel) according to the manufacturer’s instructions. mRNA was directly isolated from root hair samples using a Dynabeads mRNA DIRECT kit (Thermo Fisher Scientific) according to the manufacturer’s instructions. RNA quality was assessed on an Agilent 2100 Bioanalyser and samples were sent to GATC-Biotech^1^ for library preparation and sequencing. Sequencing data is deposited at the Short Read Archive with the BioProject ID SRP127678. Sequencing data for roots and shoots 3 dpi with R7A was obtained previously (Munch et al., 2018) and is deposited at the Short Read Archive with the BioProject ID PRJNA384655.

### Bioinformatics and Statistical Analysis

Reads were mapped to the *L. japonicus* v. 3.0 genome and differential gene expression was analyzed using CLC Genomics Workbench 9.5.3 (Qiagen). For each sample a minimum of 30 million reads were obtained with >90% of reads mapped to the reference genome. A criterion of fourfold change with a FDR *p*-value ≤ 0.05 was applied to determine differential gene expression. Results of the analysis are available on *Lotus* Base^2^ (Mun et al., 2016). Multidimensional scaling (MDS) analysis was performed in R (R Core Team, 2017) from total read count data using the script outlined in **Supplementary Data 1**. Heatmaps were generated in R using Heatmap and Heatmap3 functions (Zhao et al., 2014), an example script is provided in **Supplementary Data 1**. Venn diagrams were constructed using BioVenn (Hulsen et al., 2008). Detection of differentially expressed genes was also performed using the R package DEGseq (Wang et al., 2010) with a criterion of fourfold change with a FDR *p*-value ≤ 0.05 to examine the effects of combining replicates from different time points.

## Results

### Tissue-Specific Responses of *Lotus japonicus* to Its Compatible Symbiont *Mesorhizobium loti* R7A

A comprehensive transcriptome response of *L. japonicus* ecotype Gifu to its compatible symbiotic partner *M. loti* R7A was obtained through RNA-seq analysis of specific tissues at various stages of symbiosis. Initial interactions between rhizobia and the legume were investigated through the isolation of root hairs following R7A inoculation at 1 and 3 dpi. The NF-deficient strain R7A*nodC* was included to reveal the role of NF in these early responses, which was further examined through the isolation of purified NF-treated root hairs 2 days post-treatment. Root hair deformations of *L. japonicus* are evident as early as 24 h following inoculation with *M. loti* or treatment with purified NF (Radutoiu et al., 2003; Maekawa-Yoshikawa and Murooka, 2009). Transcriptome responses during nodule colonization were examined through the harvesting of 7 dpi nodule primordia that are in the early stages of colonization by R7A and mature nitrogen-fixing nodules that are fully colonized by R7A. RNA-seq of whole roots (minus root tips) and shoots collected at 3 dpi to provide a basis for tissue-specific responses was previously performed (Munch et al., 2018). For each condition examined, biological triplicate samples were harvested and total RNA was isolated. The reads were mapped to the *L. japonicus* v3.0 genome and all data is available from the *Lotus* Base resource website (Mun et al., 2016)^2^.

**FIGURE 1:**
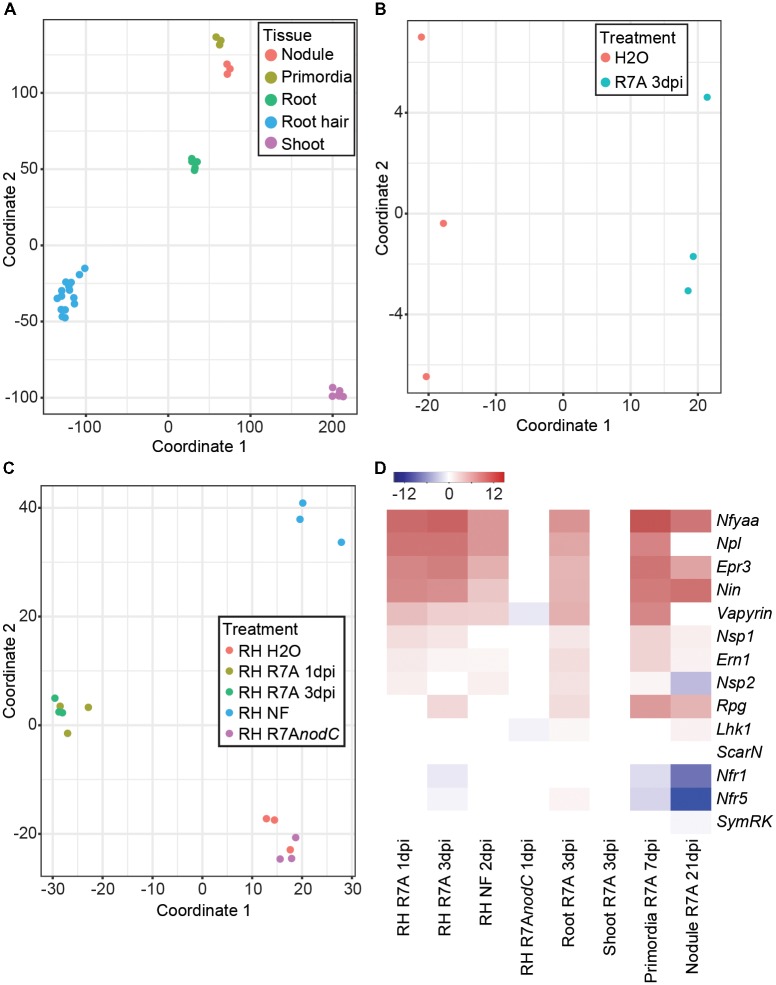
*Lotus japonicus* tissue-specific responses to the compatible symbiont *Mesorhizobium loti* R7A. **(A)** MDS plot of *L. japonicus* tissue samples. **(B)** MDS plot of whole root samples following treatment with H_2_O or R7A 3dpi. **(C)** MDS plot of all root hair samples following the indicated treatments. **(D)** Heatmap representation of log_2_ fold changes of various known symbiotic genes in tissues following treatment compared to H_2_O controls. NF, Nod factor; RH, root hairs.

Multidimensional scaling analysis clearly separated the samples based on their tissue origin (**Figure 1A**). A closer examination of whole root samples revealed a clear shift following R7A inoculation (**Figure 1B**). Most root hair samples separated into distinct groups depending on their treatment (**Figure 1C**). H_2_O and R7A*nodC* samples clustered together, indicating that the majority of early root hair transcriptome responses to R7A are dependent on NF production. R7A treated root hairs formed a cluster with separation between the 1 and 3 dpi samples, while NF-treated root hairs formed a distinct cluster removed from the R7A inoculated samples.

The expression of a set of known symbiotic genes was examined in the different tissues in response to inoculation with R7A or NF treatment compared to H_2_O-treated controls (**Figure 1D**). No difference in gene expression was observed in shoots 3 dpi with R7A or root hairs 1 dpi with the R7A*nodC* mutant compared to their respective controls, 3 dpi H_2_O-treated shoots and 3 dpi H_2_O-treated root hairs. Similar expression patterns were observed for R7A and NF treated root hair and root samples compared to control 3 dpi H_2_O-treated root hairs. Symbiotic transcription factors, *Nin* (Schauser et al., 1999), *NfyaA1* (Soyano et al., 2013), and *Ern1* (Cerri et al., 2017; Kawaharada et al., 2017a; Yano et al., 2017) were strongly induced while *Nsp1* and *Nsp2* (Heckmann et al., 2006; Kawaharada et al., 2017a) showed only slight induction. The EPS receptor *Epr3* (Kawaharada et al., 2015, 2017b), pectate lyase *Npl* (Xie et al., 2012) and *Lotus* homologs of *Vapyrin* (Murray et al., 2011) and *Rpg* (Arrighi et al., 2008) that were identified as required for IT formation in *Medicago* were all strongly induced. Notably, *Rpg* was not induced until 3 dpi with R7A. The receptors involved in NF perception, *Nfr1*, *Nfr5*, and *Symrk* (Stracke et al., 2002; Radutoiu et al., 2003) showed little change in expression other than a slight downregulation in root hairs 3 dpi.

Nodule primordia, compared to control 3 dpi H_2_O-treated roots, shared a similar expression pattern to root hair and root samples with the exception that the NF receptors *Nfr1* and *Nfr5* were downregulated. This downregulation of the NF receptors was stronger in the 21 dpi mature nodule samples compared to control 3 dpi H_2_O-treated roots. The *Nsp2* transcription factor also showed downregulation in mature nodules and expression of *Epr3*, *Npl*, and *Vapyrin* was reduced compared to primordia, root hairs, and roots.

### Early Symbiotic Responses Are Enhanced in Root Hairs and Largely Depend on Nod Factor Signaling

**FIGURE 2:**
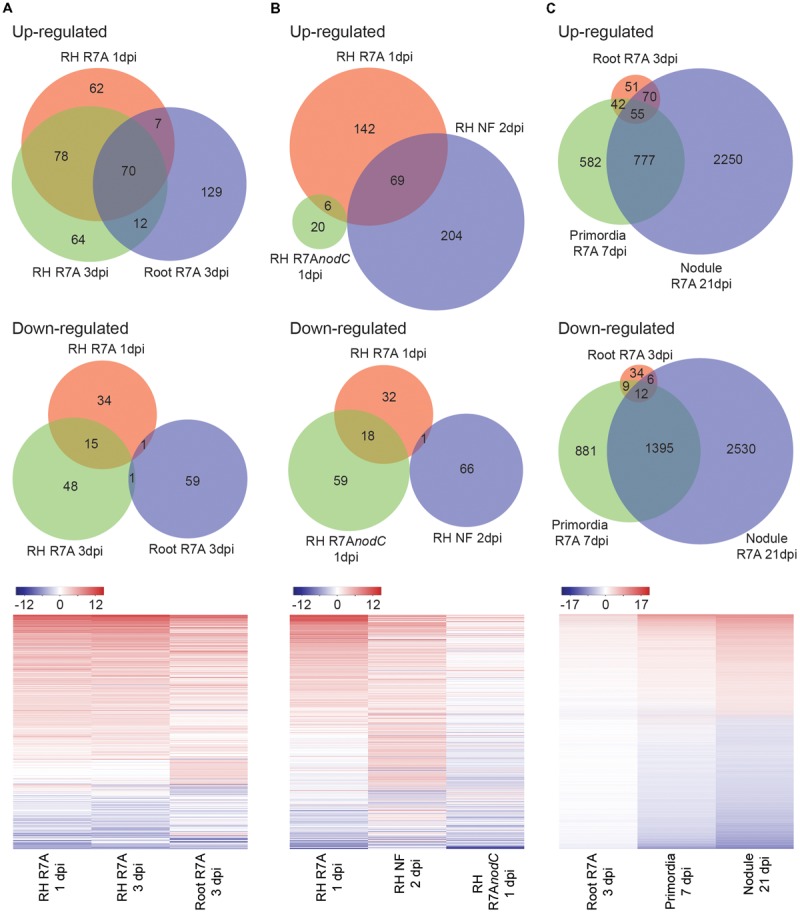
*Lotus japonicus* tissue-specific responses to compatible *M. loti* R7A and Nod factor. Venn diagram and heatmap representation of differential gene expression in the indicated conditions compared to H_2_O controls. Significant gene expression differences were designated based on a fourfold change in expression with a FDR *p*-value ≤ 0.05. **(A)** R7A induced gene expression in whole roots and root hairs. **(B)** NF-dependency of early root hair differential gene expression. **(C)** R7A induced gene expression in nodule primordia and mature nodules compared to whole roots. For heatmaps, Log_2_ fold change values were used. NF, Nod factor; RH, root hairs.

Predictably, significant overlap was observed in differentially expressed genes between root hairs harvested at 1 and 3 dpi compared to control 3 dpi H_2_O-treated root hairs and whole roots at 3 dpi compared to control 3 dpi H_2_O-treated whole roots (**Figure 2A**). Although expected symbiotic gene induction was observed in both tissues, the root hair samples showed enhanced transcriptional responses compared to whole roots (**Figure 1D** and **Supplementary Figure 1**). Among the most highly induced of the 93 genes identified as specifically differentially expressed within root hairs at 1 and 3 dpi compared to whole roots, we identified an aspartic protease and a calmodulin-binding protein as well as several blue copper proteins, expansins and pectinesterase/pectinesterase inhibitors (**Supplementary Table 2**). The root hair transcriptional changes observed in response to R7A are largely NF-dependent with root hairs inoculated with the NF-deficient R7A*nodC* strain sharing minimal overlap with R7A or NF treated samples (**Figure 2B**). Nodule primordia and mature nodules show a large transcriptional response compared to 3 dpi root samples with downregulation of genes more prominent than in other tissues investigated (**Figure 2C**).

Altogether, the tissue transcriptome data of *L. japonicus* responses to R7A provided here represents an important resource for future research into this determinate nodulating model system.

### *Lotus japonicus* Transcriptome Responses to a Spectrum of Interacting Bacteria

**FIGURE 3:**
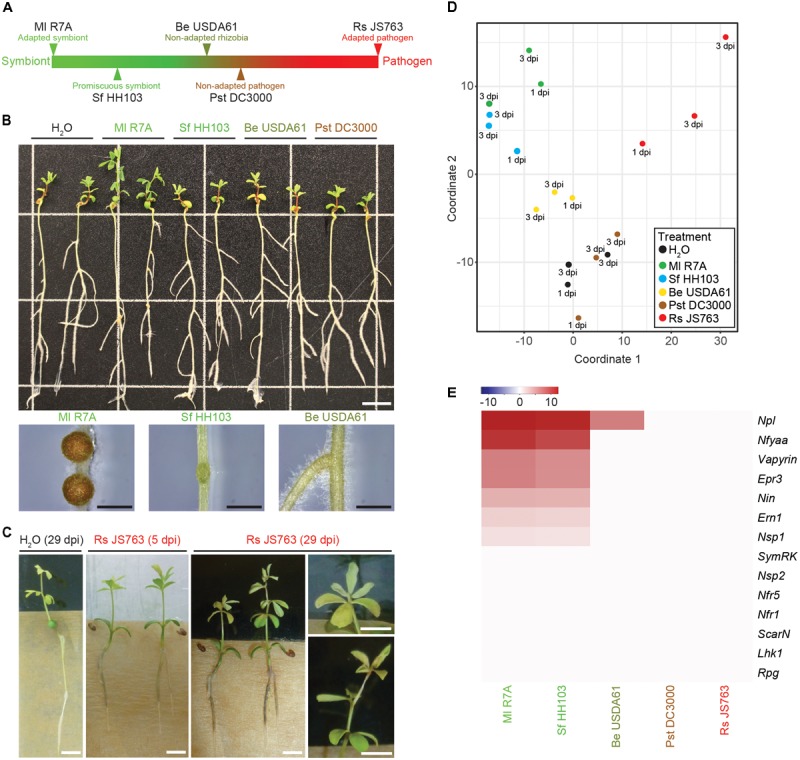
*Lotus japonicus* responses to a spectrum of interacting bacteria ranging from compatible symbiont to pathogen. **(A)** Schematic representation of the positioning of investigated bacteria on a spectrum from compatible symbiont to pathogen. **(B)** Phenotypes of *L. japonicus* 21 dpi with the indicated strains. Close up images show the differences in nodule development induced by Ml R7A, Sf HH103, and Be USDA61. Scale bars are 1 cm in the whole plant image and 1 mm in nodule images. **(C)** Representative images of root-browning and shoot wilting pathogenic phenotypes caused by Rs JS763 inoculation of *L. japonicus* at 5 and 29 dpi. Scale bars are 5 mm. **(D)** MDS plot of whole root samples following treatment with the indicated strains. **(E)** Heatmap representation of log_2_ fold changes of various known symbiotic genes following inoculation with the indicated strains compared to H_2_O controls.

Transcriptional responses of *L. japonicus* to interacting bacteria representing a spectrum from compatible symbiont to pathogen were investigated to determine similarities or differences in the host transcriptome response to these diverse bacteria (**Figure 3A**). *M. loti* R7A (Ml R7A) represents an adapted symbiont that forms nitrogen-fixing nodules on *L. japonicus*. *Sinorhizobium fredii* HH103 (Sf HH103) is a broad host-range rhizobium that forms nitrogen-fixing nodules of some species of *Lotus* but induces uninfected primordia on *L. japonicus* (Acosta-Jurado et al., 2016) and (**Figure 3B**). *Bradyrhizobium elkanii* USDA61 (Be USDA61) is a symbiont of soybean that induces minimal responses on *L. japonicus* roots, with only an occasional root swelling near lateral root junctions observed (**Figure 3B**). *Pseudomonas syringae* pv. *tomato* DC3000 (Pst DC3000) is a well-known foliar phytopathogen (Xin and He, 2013). We observed no responses to root inoculation with Pst DC3000 indicating that the strain is non-pathogenic to *L. japonicus* Gifu, as has been previously reported (Bordenave et al., 2013). *Ralstonia solanacearum* causes bacterial wilt in over 200 plant species and represents one of the most damaging bacterial pathogens in plants (Hayward, 1991). We observed that Rs JS763 causes brown discoloration of the roots from 3 dpi followed by discoloration at the base of the hypocotyl as well as chlorosis and wilting symptoms on leaves in *L. japonicus* Gifu (**Figure 3C**). These are typical plant disease symptoms due to *R. solanacearum* infection, confirming it to be a genuine root pathogen of *L. japonicus*, as previously described (Nagata et al., 2008).

Whole roots (minus root tips) treated with the spectrum of diverse bacterial strains or water were harvested at 1 and 3 dpi in biological duplicates. RNA isolation, Illumina sequencing and read mapping were performed as described above for the tissue samples. All data are available from the *Lotus* Base resource website (Mun et al., 2016)^2^. We detected outlier replicates for each inoculum and these were removed before proceeding with downstream analysis. The remaining three datasets, consisting of two 3 dpi and one 1 dpi samples, were combined to allow robust detection of differentially expressed genes. The effects of combining replicates from two 3 dpi and one 1 dpi samples were investigated using the R package DEGseq (Wang et al., 2010), which allows for differential gene expression analysis to be performed with fewer than three replicates. Differentially expressed genes were identified using the combined three replicates as well as using just the two 3 dpi replicates. A strong overlap in the genes identified between the three and two replicate analysis was observed. Almost all genes identified using the combined three replicates in our CLC genomics workbench analysis were also identified as differentially expressed in both the two and three replicate analyses using DEGseq, indicating that they likely represent genuine differentially regulated genes (**Supplementary Data 2**). However, genes oppositely regulated at 1 and 3 dpi or differentially expressed at only one time point may not have been detected due to the combined analysis of 1 and 3 dpi time points. We used the conservative lists of differentially expressed genes obtained from CLC genomics workbench in the downstream analyses. MDS analysis clustered Ml R7A and Sf HH103 together, Pst DC3000 clustered with H_2_O while Be USDA61 was slightly removed from them and Rs JS763 samples formed a less defined group removed from the other samples (**Figure 3D**).

To assess the symbiotic response induced by the diverse strains, expression of the same set of known symbiotic genes analyzed in the various tissue samples following inoculation with R7A or NF was examined through comparing bacteria inoculated root samples to control H_2_O-treated roots (**Figure 3E**). Ml R7A and Sf HH103 induced similar responses that are comparable to those obtained in analysis of the various tissues examined following R7A or NF treatment (**Figure 1D**) with the exception of *Rpg* expression, which was not induced in the Ml R7A and Sf HH103 samples. Only the *Npl* gene showed any expression change following inoculation with Be USDA61 and no response for any of the genes was observed following Pst DC3000 or Rs JS763 inoculation.

### *L. japonicus* Responses to a Non-adapted Pathogen and Incompatible Rhizobia

**FIGURE 4:**
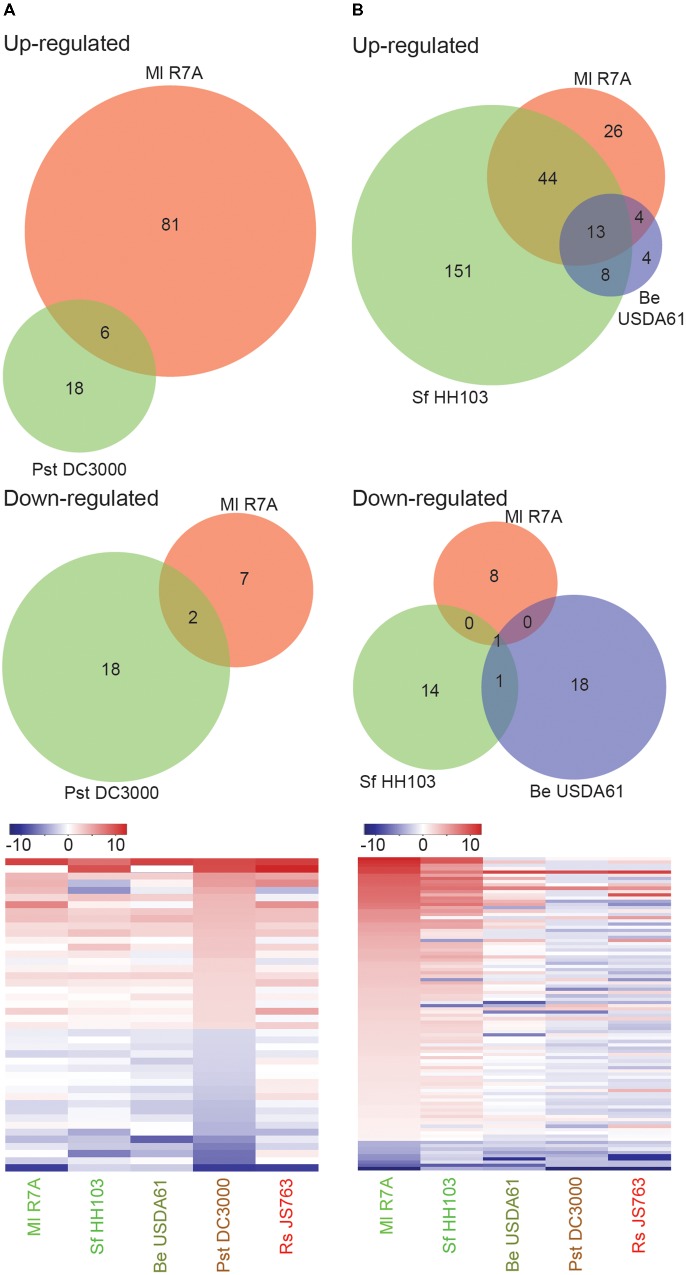
*Lotus japonicus* root responses to non-adapted pathogenic bacteria and incompatible rhizobia. Venn diagram and heatmap representations of differential gene expression in the indicated conditions compared to H_2_O controls. Significant gene expression differences were designated based on a fourfold change in expression with a FDR *p*-value ≤ 0.05. **(A)** Non-adapted pathogen Pst DC3000 induced differential gene expression. **(B)** Compatible symbiont Ml R7A compared to incompatible rhizobia Sf HH103 and Be USDA61 differential gene expression. For heatmaps, Log_2_ fold change values were used.

Pst DC3000 induced no discernible phenotypic responses on *L. japonicus*. Only a small set of genes (44) showed significant expression changes following Pst DC3000 treatment, 8 of which showed similar regulation following Ml R7A inoculation (**Figure 4A** and **Supplementary Table 3**). Similar expression of the identified Pst DC3000 differentially regulated genes was observed following inoculation with the spectrum of strains investigated, indicating that these genes may represent a common response to bacteria by *L. japonicus* (**Figure 4A** and **Supplementary Table 3**). Of interest in the identified genes are two leucine-rich repeat (LRR) receptor like proteins (Lj0g3v0095839 and Lj0g3v0331049), an unknown-conserved GYF-domain containing protein (Lj5g3v0101610) and an exportin-7 like protein (Lj1g3v2392240) that show upregulated expression in response to all of the diverse strains. LRR receptors represent the largest receptor family in plants and are involved in diverse developmental, defense and symbiotic processes (Torii, 2004). Exportin-7 proteins are reportedly involved in nuclear export processes (Mingot et al., 2004).

Be USDA61 and Sf HH103 represent two rhizobial species that are incompatible for the establishment of nitrogen-fixing symbiosis with *L. japonicus* (**Figure 3B**). Significant gene expression changes were limited following Be USDA61 inoculation, whilst Sf HH103 inoculation induced a greater response and shared a large overlap with Ml R7A inoculation (**Figure 4B**).

Genes showing significant differential expression following Ml R7A inoculation but not Sf HH103 or Be USDA61 represent candidates involved in specifically promoting interaction with the compatible symbiont. The 34 genes identified that fit these criteria are listed in **Supplementary Table 4**. Of particular interest in this list are Lj5g3v2288900 that encodes a legume specific chalcone isomerase that has recently been shown to be involved in the biosynthesis of an Ml R7A NodD1 activating inducer, specifically within ITs (Kelly et al., 2017b) and Lj4g3v2365210 that encodes for the Nfy-B1 subunit, which forms part of the Nuclear Factor Y transcriptional factor known to be involved in nodulation (Soyano et al., 2013). Lj4g3v1983610 encodes an ENOD-like protein with a predicted cupredoxin domain. The protein shares 46% amino acid identity to ENOD16 of *M. truncatula*, which has been shown to be induced during symbiosis. No clear roles for ENOD16 have been demonstrated but it has been proposed that they may be involved in cell wall reorganization (Greene et al., 1998), which is required for IT development.

### Interplay Between Symbiotic and Defense Responses in *L. japonicus*

Rs JS763 was shown to be a root pathogen of *L. japonicus* (**Figure 3B**). Rs JS763 inoculation resulted in a distinct transcription response, with 347 (277 upregulated and 70 downregulated) differentially expressed genes identified (**Supplementary Data 3**). Among the genes upregulated by Rs JS763 are five genes annotated as WRKY transcription factors (Lj0g3v0074419, Lj0g3v0244439, Lj1g3v1815540, Lj1g3v1787630, and Lj1g3v1220240). WRKY transcription factors belong to a large family of regulatory proteins involved in various plant processes but most notably in plant immunity against pathogen attack, including *R. solanacearum* infection (Mukhtar et al., 2008; Wang et al., 2013, 2014; Dang et al., 2014; Cai et al., 2015; Liu et al., 2017). Two 1-aminocyclopropane-1-carboxylate oxidase (ACO) 1-like genes (Lj2g3v0911860 and Lj6g3v0086450) were also induced. ACO proteins are involved in biosynthesis of ethylene, a plant hormone that is involved in many developmental and physiological responses including defense (Broekgaarden et al., 2015) and symbiosis (Reid et al., 2018). Ethylene signaling plays a critical role in bacterial wilt disease development in *A. thaliana* (Hirsch et al., 2002), *M. truncatula* (Moreau et al., 2014) and in tomato resistance to *R. solanacearum* (Zhang et al., 2004). In addition to inducing ethylene production by the host plant, *R. solanacearum* is also capable of producing ethylene itself (Weingart et al., 1999). Other defense related genes induced by Rs JS763 included pathogenesis-related proteins [such as PR1a: Lj6g3v2170740, beta-1,3-glucanase (PR-2): Lj0g3v0278459; PR10: Lj0g3v0286359; peroxidase 1-like: Lj0g3v0336889; two chitinases: Lj5g3v1961260 and Lj6g3v1078650] and several protease inhibitor proteins potentially involved in plant immunity (Lj0g3v0174139, Lj0g3v0174109, Lj2g3v0604160, Lj0g3v0265049, Lj5g3v1174410). Homologs of some of these genes were also reported as differentially expressed in response to *R. solanacearum* in *M. truncatula* according to proteomic and transcriptomic analyses (Yamchi et al., 2018). Interestingly, a gene annotated as a WAT1 (Walls Are Thin1)-related gene (Lj1g3v0913340) was downregulated in response to Rs JS763. In *A. thaliana*, inactivation of WAT1 gene, which is required for secondary cell-wall deposition, conferred broad-spectrum resistance to vascular pathogens, including *R. solanacearum* (Denance et al., 2013).

**FIGURE 5:**
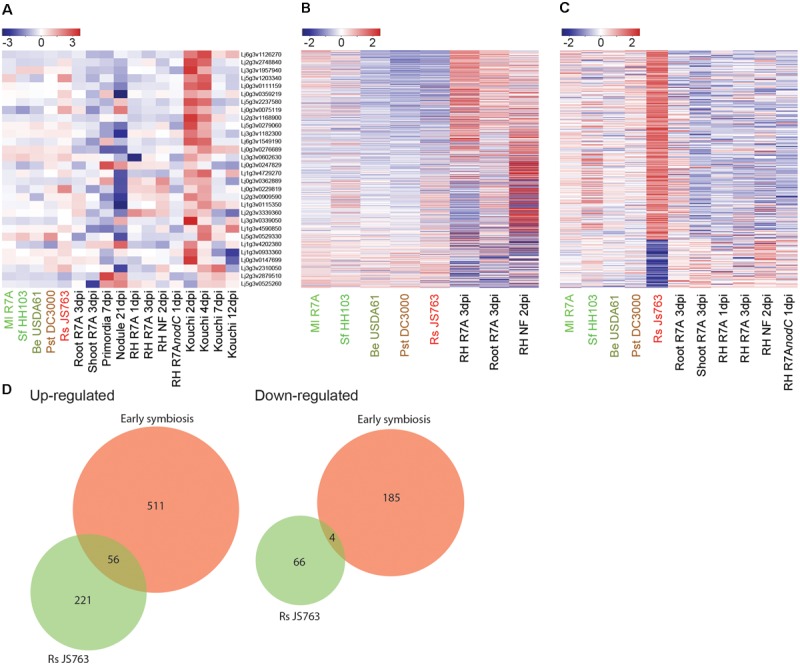
Interplay between symbiotic and defense transcriptional responses. **(A)** Log_2_ fold changes of potential defense associated genes identified as induced in the early stages of symbiosis by Kouchi et al. (2004) were examined across our datasets. **(B)** Expression patterns of 71 commonly differentially expressed genes in early symbiotic samples compiled from Ml R7A inoculated roots and root hairs was analyzed in the spectrum of interacting bacteria. **(C)** Rs JS763 differentially regulated genes were examined for expression changes in early symbiotic and diverse strain datasets. **(D)** Venn diagrams showing the overlap in gene expression between early symbiosis, corresponding to a compiled list of genes that were responsive to either Ml R7A or NF in whole roots or root hairs, and response to pathogenic Rs JS763. Differentially expressed genes were filtered by a minimum fourfold change and a FDR *p*-value ≤ 0.05.

A transient defense response in *L. japonicus* roots during the early stages of symbiosis with compatible rhizobia had previously been reported (Kouchi et al., 2004). We investigated expression of the defense related genes identified by Kouchi et al. (2004) but did not observe comparable expression in response to symbiotic or pathogenic strains in our dataset (**Figure 5A** and **Supplementary Data 4**). Since symbiosis genes were consistently induced across all our experiments, we take this as an indication that the set of genes with defense-related annotations identified by Kouchi et al. (2004), are not generally associated with symbiotic or pathogenic interactions and our data does not support the notion that an early defense-like response is evoked by compatible rhizobia in the establishment of symbiosis with *L. japonicus*.

It remains possible, however, that overlaps in gene expression responses could exist between infection-competent symbiotic and pathogenic bacteria. To further examine such potential similarities, a list of commonly differentially expressed genes in early symbiotic samples was compiled from Ml R7A inoculated roots and root hairs. Expression of the 71 genes was analyzed in the spectrum of interacting bacteria, including Rs JS763 (**Figure 5B**). In a complementary analysis, expression of the 347 differentially expressed genes following Rs JS763 inoculation was compared in response to the diverse strains (**Figure 5C**). Rs JS763 inoculated roots did not show similar gene expression patterns with the symbiotic samples in either of these analyses. However, some overlapping expression changes were observed between Rs JS763 responsive genes and a compiled list of early symbiotic genes that were responsive to either Ml R7A or NF in whole roots or root hairs (**Figure 5D** and **Supplementary Table 5**).

This overlap included the cytokinin oxidase *Ckx3* (Lj5g3v0692300) that is involved in cytokinin breakdown during nodulation (Reid et al., 2016) and the sulfate transporter *Sst1* (Lj2g3v0776860.1) that is required for nitrogen-fixation within nodules. In addition, putative defense-related genes identified were a PR10-like gene (Lj0g3v0286359), the WAT1-related gene (Lj1g3v0913340) and a gene encoding a salicylate *O*-methyltransferase-like protein (Lj6g3v0509430) that is involved in the biosynthesis of methyl salicylate which was found to participate in root responses against fungal pathogens (Ament et al., 2010; Boba et al., 2016). Further studies will be required to determine the roles of the symbiosis-related genes in the *Lotus*–*Ralstonia* interaction and of the putative defense genes in *Lotus*–rhizobium interactions. However, the identification of genes responding to both *Ralstonia* and rhizobium treatments does not necessarily indicate that symbiotic rhizobia are initially perceived as pathogenic by *Lotus*. In contrast, our data indicate that the pathogenic and symbiotic responses are well-separated as early as 1 dpi (**Figure 3D**).

## Discussion

*Lotus japonicus* has been extensively utilized for the study of symbiotic interactions with rhizobial bacteria and mycorrhizal fungi. Analysis of the host transcriptional response during these interactions has been beneficial in developing our understanding of the molecular processes behind the symbiotic interactions and identifying potential genes important for these.

Transcriptome data collected from numerous microarray based studies of *L. japonicus* tissues at various stages of development or in association with biotic and abiotic treatments has been collated together in the LjGEA and is now accessible through *Lotus* Base^2^ (Sanchez et al., 2008, 2011; Guether et al., 2009; Hogslund et al., 2009; Diaz et al., 2010; Verdier et al., 2013; Mun et al., 2016). We have expanded on this through the generation of a comprehensive RNA-seq based transcriptome analysis of the response of *L. japonicus* to its compatible symbiont *M. loti* R7A. Previous studies in *M. truncatula* have highlighted the benefits of isolating root hairs to amplify early symbiotic transcriptome responses (Breakspear et al., 2014). In our analysis, early symbiotic signaling was similarly amplified in root hair samples compared to whole roots. *Npl* (Xie et al., 2012), *Vapyrin* (Murray et al., 2011), and *Rpg* (Arrighi et al., 2008) represent genes known to be required for IT development that were highly induced in our root hair samples. Additional genes identified as showing enhanced expression within root hairs represent potential candidates that may be involved in IT development. An interesting candidate from this analysis is the aspartic peptidase APN1 (Lj3g3v0950730.1) that was recently discerned to be expressed in nodules and demonstrated to play a crucial role in nodule functioning. *apn1* mutant nodules fail to fix nitrogen and undergo early senescence in a *M. loti* strain-depended manner (Yamaya-Ito et al., 2017). Our identification of root hair enhanced expression of *Apn1* compared to whole roots suggests that IT phenotypes of *apn1* inoculated with R7A would be of interest to examine. As expected, early symbiotic signaling was largely NF-dependent and although considerable overlap was observed for differentially expressed genes following R7A or NF treatment, including most of the known symbiotic genes, there remains clear differences in the responses. A comparable difference in differentially expressed genes following NF treatment or *S. meliloti* inoculation was observed in *M. truncatula* (Breakspear et al., 2014). The observed differences in responses to purified NF compared to rhizobial inoculation may be due to the effects of other rhizobial factors, for example surface glycans, or differences in the concentration of NF roots are exposed to following rhizobial inoculation compared to purified NF treatment. Rhizobia produce an array of NF species and it is also possible that this induces differences in transcriptional responses compared to application of one particular purified NF species. Interestingly, *Rpg* induction was not observed in 2 dpi NF-treated or at 1 dpi with R7A, suggesting that *Rpg* induction is delayed compared to the other symbiotic genes investigated. This delay was not observed in *M. truncatula*, where *Rpg* induction was detected in roots at 1 dpi with *S. meliloti* or NF-treated root hairs (Arrighi et al., 2008; Breakspear et al., 2014). Nodule primordia and mature nodules exhibited large transcriptional reprogramming as has previously been observed for *Lotus* nodules (Takanashi et al., 2012). Highly upregulated genes include various transporters, auxin and cytokinin responsive genes as well as genes known to be required for nodule functioning such as the sulfate transporter *Sst1* (Krusell et al., 2005) and homocitrate synthase *Fen1* (Hakoyama et al., 2009).

Model legume resources, developed largely through the study of symbiotic interactions, have not been exploited to such an extent in interactions with diverse microbes. In this study, we identified distinct transcriptome responses to a spectrum of interacting bacteria ranging from symbiotic to pathogenic. The broad host-range rhizobium Sf HH103 forms small nodule primordia that remain uninfected on *L. japonicus* (Sandal et al., 2012; Acosta-Jurado et al., 2016). A remarkably similar early transcriptome response of *L. japonicus* to Ml R7A and Sf HH103 was observed, indicating that NF-induced signaling is comparable between the two strains. Both strains produce a variety of NFs with one notable difference being the substitution on the reducing terminal residue, which is generally an acetylfucosyl for Ml R7A NF and a methylfucosyl for Sf HH103 NF (Gil-Serrano et al., 1997; Rodpothong et al., 2009). Genes identified as potentially limiting the symbiotic capacity of Sf HH103 based on differential expression compared to Ml R7A include a legume specific chalcone isomerase, Nfy-B1 and an ENOD16 encoding gene. Be USDA61 induced only occasional root swellings on *L. japonicus* and a limited transcriptome response was observed, suggesting that the NF species produced by this rhizobial strain are not well-perceived by *L. japonicus*. We also detected minimal transcriptome responses to Pst DC3000 inoculation, which appeared to be non-pathogenic to *L. japonicus* roots. Differentially expressed genes identified from these non-symbioitic/non-pathogenic strains may represent a common response to bacteria as similar transcription responses were overserved across the spectrum of interacting bacteria.

Rs JS763 was characterized as a genuine pathogen of *L. japonicus* with typical disease symptoms observed following root inoculation and a clear and distinct root transcriptome response was detected, allowing us to investigate the interplay between pathogenic and symbiotic signaling. Transcriptome-based studies have previously suggested that an initial defense-like response to rhizobia is subsequently dampened through symbiotic signaling in soybean/*B. japonicum*, *M. truncatula*/*S. meliloti*, and *L. japonicus*/*M. loti* symbioses (Kouchi et al., 2004; Lohar et al., 2006; Libault et al., 2010). Kouchi et al. (2004) identified a set of potential defense-related genes that were induced at early time points before being suppressed at later stages of *L. japonicus*/*M. loti* interactions. We did not observe similar induction of the identified genes in our RNA-seq transcriptome analysis following *M. loti* R7A inoculation, indicating that the gene set is not reproducibly associated with symbiotic infection. Furthermore, only two of these genes (Lj0g3v0229819 and Lj5g3v1203340) encoding carboxylesterase 6-like and basic 7S globulin-like proteins respectively, showed a significant response following Rs JS763 inoculation in our analysis, suggesting that although the genes identified by Kouchi et al. (2004) have potential defense associated annotations they may not represent genes that are generally pathogen responsive in *L. japonicus*. It is possible that differences in host and *M. loti* genotypes as well as growth setups used in our analysis and by Kouchi et al. (2004) may be responsible for the differences in transcriptional responses observed.

We did however identify a small overlap between early time point Ml R7A responsive genes and Rs JS763 differentially expressed genes. The only known symbiotic genes identified as commonly transcriptionally regulated are the cytokinin oxidase *Ckx3* and the sulfate transporter *Sst1*. Cytokinin is a key plant hormone and is tightly regulated in root development and symbiotic processes (Reid et al., 2017). Transcriptional changes in the expression of cytokinin related genes have previously been reported following *R. solanacearum* inoculation of *M. truncatula* (Moreau et al., 2014). SST1 functions during the later stages of symbiosis to transport sulfate from the plant to rhizobia where it is essential for protein synthesis, including nitrogenase biosynthesis (Krusell et al., 2005). It is unclear if a similar role is performed by SST1 in earlier stages of interactions with rhizobia or other bacteria. The only obvious potentially defense-related genes identified in the list of commonly induced genes were a PR10-like gene (Lj0g3v0286359), WAT1-related gene (Lj1g3v0913340) and a gene encoding a salicylate *O*-methyltransferase-like protein (Lj6g3v0509430). Lj6g3v0509430 is one of several salicylate *O*-methyltransferase-like proteins with high similarity encoded by *L. japonicus* and additional studies are required to determine if it is involved in salicylic acid responses.

Altogether, our analysis of transcriptome responses to a spectrum of interacting bacteria indicates that distinct transcriptome responses are observed in response to symbiotic and pathogenic bacteria and does not support the concept that an early defense-like response is generally evoked by compatible rhizobia in *L. japonicus* during the establishment of symbiosis.

## Author Contributions

SK and TM performed the plant experiments, isolated RNA, and analyzed the sequencing data. SUA conceived and supervised the study. CB and JS devised the experiments and contributed resources. SK, CB, and SUA wrote the manuscript with input from all authors.

## Conflict of Interest Statement

The authors declare that the research was conducted in the absence of any commercial or financial relationships that could be construed as a potential conflict of interest.

## Footnotes

1 https://gatc-biotech.com

2 https://lotus.au.dk
