# Supplementary material for: Distinct Lotus japonicus Transcriptomic Responses to a Spectrum of Bacteria Ranging From Symbiotic to Pathogenic
Source: Front Plant Sci. 2018 Aug 20;9:1218. doi: 10.3389/fpls.2018.01218 (PMC6110179; doi:10.3389/fpls.2018.01218)
Supplement: Supplementary file 1 [file Image_1.PDF]

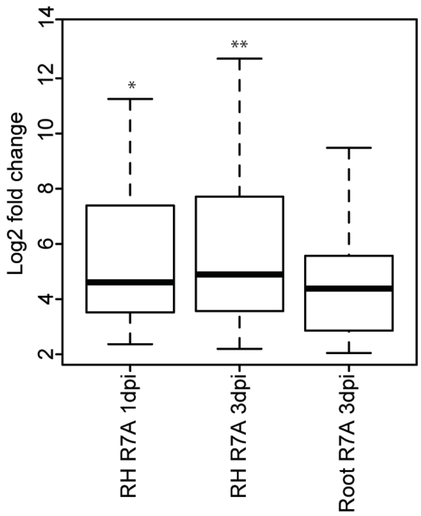

Supplemental Figure 1. Enhanced expression of symbiotically induced genes in root hairs. Boxplot of log2 fold change expression values of genes commonly induced in roots 3 dpi and root hairs at 1 and 3 dpi. Asterisks indicate significant differences in the average log2 fold change of the root hair samples compared to whole roots (t. tests, p-value *=0.05, **=0.01).
