# Supplementary material for: Distinct Lotus japonicus Transcriptomic Responses to a Spectrum of Bacteria Ranging From Symbiotic to Pathogenic
Source: Front Plant Sci. 2018 Aug 20;9:1218. doi: 10.3389/fpls.2018.01218 (PMC6110179; doi:10.3389/fpls.2018.01218)
Supplement: Supplementary file 2 [file Table_1.PDF]

Supplemental Table 1. Bacterial strains

Bacteria	Relevant characteristics	Reference
M. loti R7A	*L. japonicus* symbiont	(Sullivan et al., 2002;Kelly et al., 2014)
M. loti R7A*nodC*	NF-deficient R7A strain due to *nodC* markerless deletion	(Rodpothong et al., 2009)
S. fredii HH103	Incompatible *L. japonicus* rhizobia	(Sandal et al., 2012;Acosta-Jurado et al., 2016)
B. elkanii USDA61	Incompatible *L. japonicus* rhizobia, Pol (50 ug/mL^{-1})	(Okazaki et al., 2013)
P. syringae DC3000	Plant pathogen, Rif (50 ug/mL^{-1}), Kan (50 ug/mL^{-1})	(Buell et al., 2003)
R. solanacearum JS763	*L. japonicus* pathogen	(Poussier et al., 2000;Vailleau et al., 2007)
