# Supplementary material for: Distinct Lotus japonicus Transcriptomic Responses to a Spectrum of Bacteria Ranging From Symbiotic to Pathogenic
Source: Front Plant Sci. 2018 Aug 20;9:1218. doi: 10.3389/fpls.2018.01218 (PMC6110179; doi:10.3389/fpls.2018.01218)
Supplement: Supplementary file 3 [file Table_2.PDF]

Supplemental Table 2. Root hair specific responses to R7A inoculation

Name	Gene annotation	RH R7A 1dpi	FDR p-value	RH R7A 3dpi	FDR p-value	RH nodC 1dpi	FDR p-value	RH NF 2dpi	FDR p-value	Root R7A 3dpi	FDR p-value
Lj3g3v0950730	aspartic proteinase CDR1-like	8.14	0.02	9.58	1.71E-03	NaN	NaN	NaN	NaN	0.16	1
Lj0g3v0169829	blue copper protein-like	10.36	4.47E-04	8.57	8.19E-03	NaN	NaN	6.26	0.21	4.89	0.55
Lj4g3v2618530	multifunctional transport intrinsic membrane protein 2	8.38	0.01	8.26	0.01	NaN	NaN	4.91	0.54	7.58	0.07
Lj2g3v1014120	MtN24-like	7.73	0.03	7.8	0.02	NaN	NaN	1.55	0.93	1.88	7.25E-03
Lj3g3v0323320	calmodulin-binding family protein isoform 1	4.63	0.02	7.1	1.54E-06	-2.01	1	1.55	0.91	1.44	0.02
Lj4g3v2704690	basic blue protein	4.32	0	6.79	0	0.26	1	2.8	6.05E-12	1.71	0
Lj1g3v3975740	blue copper protein-like	7.6	1.34E-07	6.31	4.87E-05	-0.17	1	3.6	0.19	1.27	0.43
Lj1g3v4288790	hypothetical protein	4.96	0	6.05	0	-0.26	1	2.67	7.81E-10	1.87	0
Lj2g3v1415140	hypothetical protein	4.72	0	5.62	0	0.16	1	2.49	0	1.11	0
Lj0g3v0119529	multidrug and toxin extrusion protein 2-like	5.69	1.05E-06	5.54	2.13E-06	0.33	1	3.72	0.15	1.36	0.01
Lj5g3v2292490	protoheme IX farnesyltransferase, mitochondrial-like	4.36	9.36E-05	5.4	4.50E-07	5.58	3.88E-06	-1.35	0.93	-0.16	0.77
Lj2g3v2598860	expansin	5.91	0	5.14	0	1.18	1	5.14	0	1.61	0
Lj0g3v0188379	pectinesterase/pectinesterase inhibitor PPE8B-like	5.14	0	5.11	0	-0.99	0.94	3.42	0	1.82	0
Lj2g3v3339140	polygalacturonase	5.85	0	4.8	2.75E-13	1.56	0.71	5.32	0	1.55	0
Lj4g3v2215400	probable pectinesterase/pectinesterase inhibitor 12-like	5.23	1.04E-09	4.8	3.92E-08	-0.19	1	6.1	3.97E-04	1.16	1.60E-09
Lj0g3v0128629	trichome birefringence-like 27	4.22	0.05	4.65	0.01	0.61	1	0.73	0.99	0.44	1
Lj0g3v0013599	expansin	4.69	8.90E-06	4.55	1.17E-05	0.19	1	4.27	1.01E-06	1.88	1.72E-05
Lj1g3v3329770	bidirectional sugar transporter N3-like	3.16	3.58E-11	4.45	0	-0.74	1	1.62	0.24	0.03	1
Lj1g3v3317470	cytokinin hydroxylase-like	4.82	2.29E-11	4.33	9.51E-09	1.17	1	4.34	1.85E-12	0.6	0.06
Lj2g3v1415130	hypothetical protein	3.71	2.40E-13	4.28	0	-0.47	1	1.05	0.82	0.75	8.79E-03
Lj0g3v0154359	adenylate isopentenyltransferase	4.89	1.29E-11	4.2	4.44E-08	0.51	1	1.96	0.08	-0.17	0.99
Lj0g3v0132199	non-haem dioxygenase, SRG1-like	2.99	2.51E-04	4.17	2.37E-11	0.43	1	1.79	0.03	1.59	8.63E-05
Lj3g3v0730030	BAHD acyltransferase At5g47980-like, partial	2.84	0.03	4.12	1.46E-06	1.87	0.59	-0.15	1	1.05	4.09E-03
Lj2g3v1226690	uncharacterised protein	4.12	9.83E-09	4.11	6.09E-10	0.37	1	1.53	0.34	0.74	0.17
Lj3g3v1354750	Cysteine-rich repeat secretory protein	3.44	1.63E-13	4.09	0	0.84	1	0.58	0.79	0.73	0.02
Lj0g3v0299009	uncharacterised protein	4.36	0	4.03	0	0.03	1	2.06	3.07E-03	1.13	9.66E-06
Lj5g3v0055650	hypothetical protein	5.15	2.75E-11	4.02	6.34E-03	1.13	1	-5.52	0.25	NaN	NaN
Lj1g3v4024010	protein TRANSPARENT TESTA	2.28	0.04	3.98	8.82E-09	-0.03	1	1.73	0.28	1.19	2.31E-09
Lj0g3v0078039	exocyst complex component	4.3	1.38E-05	3.91	8.72E-04	3.1	0.12	-0.42	1	1.76	0.15
Lj1g3v3704730	auxin-induced protein 5NG4-like	3.45	5.67E-05	3.91	1.14E-07	-0.65	1	1.36	0.66	0.28	0.96
Lj6g3v0574840	hypothetical protein	3.65	0.03	3.86	0.02	3.41	0.04	2.73	0.31	6.35	0.26
Lj0g3v0342759	uncharacterised protein	4.53	1.47E-07	3.86	6.14E-04	4.07	4.79E-06	2.49	0.43	1.19	0.92
Lj4g3v0473620	E6-like protein	3.5	0	3.8	0	0.24	1	2.49	0	0.55	0.03
Lj1g3v0183010	Thioredoxin superfamily protein	4.79	0	3.79	0	-0.87	0.53	3.46	0	1.8	0
Lj6g3v2170750	pathogenesis-related protein	4.32	0	3.78	0	-0.22	1	5.62	0	1.35	4.64E-13
Lj1g3v1784380	hypothetical protein	2.18	6.68E-03	3.64	1.46E-11	-0.53	1	-0.02	1	-1.48	5.32E-09
Lj4g3v1327480	subtilase	3.84	0	3.59	0	-0.15	1	1.95	3.81E-09	-0.25	0.71
Lj5g3v1170930	ankyrin repeat-containing protein At3g12360-like	3.4	1.07E-06	3.52	1.62E-08	1.32	0.75	0.44	0.99	0.52	0.02
Lj3g3v2261380	probable sulfate transporter 3.4-like	3.48	0	3.49	0	-0.34	1	2.09	2.05E-03	0.27	0.17
Lj2g3v0813080	rac-like GTP-binding protein ARAC7-like	2.47	7.97E-05	3.37	1.86E-12	0.22	1	1.63	0.14	0.37	0.21
Lj1g3v4918670	epoxide hydrolase 2-like	3.77	0	3.35	0	0.18	1	1.57	4.91E-04	0.2	0.52
Lj5g3v2013610	Indole-3-acetic acid-amido synthetase GH3.3	3.5	0	3.26	1.06E-11	-0.36	1	2.77	0.01	1	3.66E-04
Lj2g3v1925800	PAR-1a protein	4.57	0	3.26	0	0.72	0.91	5.26	0	0.68	1.41E-08
Lj3g3v3500200	gibberellin 2-beta-dioxygenase 8-like	4.84	2.76E-06	3.19	0.01	-3.31	1	5.09	0.45	1.06	0.11
Lj3g3v1342650	Cysteine-rich repeat secretory protein	2.55	6.42E-14	3.13	0	0.59	0.97	1.12	0.12	1.34	4.38E-11
Lj1g3v4918680	epoxide hydrolase	3.48	0	3.12	0	3.94E-03	1	1.9	9.02E-12	0.18	0.63
Lj3g3v1663540	transcription factor bHLH30-like	2.78	0	3.06	0	-0.55	0.99	1.03	0.09	0.64	5.35E-08
Lj1g3v0050960	pentatricopeptide repeat-containing protein	2.97	4.53E-04	3	1.08E-04	1.74	0.77	-0.8	0.65	-0.14	1
Lj0g3v0188889	cytochrome P450	4.59	4.65E-07	2.99	0.03	1.65	0.9	-0.25	1	2.17	0.27
Lj5g3v0525250	chitotriosidase-1-like	2.85	8.06E-10	2.97	1.91E-10	1.05	0.72	0.03	1	0.72	8.87E-10
Lj0g3v0348489	pathogenesis-related protein PR10	2.05	0	2.95	0	-0.54	0.82	0.94	0.09	1.64	0
Lj1g3v4918750	epoxide hydrolase 2-like	3.2	0	2.84	0	0.01	1	2.37	8.97E-08	0.09	1
Lj2g3v0855300	retinol dehydrogenase 14-like isoform 1	2.21	1.11E-08	2.78	4.26E-14	0.19	1	2.05	7.30E-07	0.91	0
Lj1g3v0342000	probable RNA-dependent RNA polymerase 5-like	2.6	0	2.78	0	-0.17	1	0.81	0.02	0.6	1.29E-06
Lj5g3v0465980	thermospermine synthase ACAULIS5-like	3.41	0	2.77	5.85E-10	0.71	1	1.32	0.08	1.41	0
Lj2g3v1757060	uncharacterised protein	3.32	2.76E-06	2.66	1.01E-03	-0.35	1	4.26	0.06	1.12	0.44
Lj2g3v2560080	gibberellic acid-stimulated protein 1	2.83	0	2.65	0	0.44	1	3.69	0	0.45	1.07E-03
Lj0g3v0188899	cytochrome P450	3.88	0	2.63	9.78E-08	0.51	1	1.22	0.28	1.57	3.36E-03
Lj1g3v3716810	wall-associated receptor kinase 2-like	2.79	4.28E-05	2.58	2.76E-05	-0.79	1	0.47	0.96	0.57	0.45
Lj2g3v0776860	chalcone synthase 1	3.17	3.25E-06	2.58	1.48E-03	-0.19	1	0.22	1	0.82	0.54
Lj0g3v0290889	sst1 protein	2.09	2.00E-05	2.58	0	-1.08	0.32	2.35	3.90E-04	1.19	0
Lj1g3v4875690	probable pectinesterase/pectinesterase inhibitor 41-like	2.27	0	2.57	0	0.3	1	0.84	0.1	1.06	2.61E-11
Lj3g3v1307410	uncharacterised protein	3.52	0	2.52	3.50E-10	0.26	1	2.1	5.55E-05	1.22	1.41E-05
Lj1g3v2126120	mannan endo-1,4-beta-mannosidase 7-like	2.81	0	2.51	0	-0.15	1	1.1	7.71E-04	0.74	7.52E-10
Lj0g3v0275539	ubiquitin carboxyl-terminal hydrolase-like	2.11	0	2.46	0	-0.07	1	0.52	0.11	1.31	0
Lj2g3v1925790	copper transporter 6-like isoform 1	2.7	3.43E-03	2.45	0.02	-0.65	1	1.06	0.83	1.01	0.59
Lj0g3v0091689	Indole-3-acetic acid-amido synthetase GH3.3	2.7	0	2.45	0	-1.16	4.60E-03	2.5	4.87E-14	0.6	7.89E-04
Lj0g3v0201489	uncharacterised protein	2.87	0	2.39	0	-0.48	0.95	2.53	1.46E-09	1.82	0
Lj0g3v0168319	MFS transporter	3.17	0	2.37	0	0.2	1	0.61	0.12	1.93	0
Lj1g3v4729110	LOG1	2.37	4.85E-05	2.32	5.10E-05	0.21	1	1.09	0.3	1.22	3.63E-04
Lj1g3v4918700	epoxide hydrolase 2-like	2.07	3.14E-04	2.29	1.52E-05	-0.64	1	1.64	0.03	-0.36	0.19
Lj2g3v0911840	transcription factor MYB1R1	2.08	1.29E-05	2.29	5.13E-09	-0.16	1	0.29	0.97	1.76	2.72E-08
Lj6g3v1618120	ent-kaurenoic acid oxidase 2-like	2.13	7.55E-12	2.23	0	-0.46	1	1.2	1.35E-04	0.45	5.99E-04
Lj6g3v1177370	subtilisin-like protease	2.08	0	2.21	0	0.3	1	0.98	9.18E-06	0.15	0.98
Lj3g3v0786210	beta-glucosidase 12-like	2.54	0	2.18	0	-0.19	1	0.8	0.06	0.71	1.56E-05
Lj2g3v0322770	probable receptor-like protein kinase	2.08	0.03	2.09	0.02	0.38	1	0.21	1	0.09	1
Lj0g3v0266379	cytochrome P450 93A3-like	2.29	1.71E-11	2.04	1.24E-08	-0.52	1	2.61	4.99E-06	-0.03	1
Lj3g3v2575540	pectinesterase 3-like	2.61	0	2.01	0	-0.28	1	1.83	2.64E-07	0.68	1.77E-08
Lj5g3v0243320	uncharacterised protein	-2.12	0	-2.01	0	-2.42	0	1.78	3.01E-07	-1.96	0.09
Lj2g3v0632430	glutamate decarboxylase-like	-2.08	3.47E-11	-2.02	1.47E-10	-0.31	1	0.29	0.97	-0.34	0.04
Lj6g3v1319720	uncharacterised protein	-2.02	0	-2.29	0	-1.09	4.54E-07	0.36	0.8	-2.01	0.06
Lj4g3v3056170	Alpha/beta-Hydrolases superfamily protein	-3.03	1.36E-06	-2.62	2.94E-06	-1.83	1.74E-03	-0.02	1	-0.18	1
Lj1g3v4317620	seed maturation protein LEA 4	-4.88	0	-2.71	8.96E-04	-1.23	9.82E-03	1.07	0.8	0.48	0.59
Lj3g3v2225730	late embryogenesis abundant protein 1-like	-3.61	0	-2.73	1.24E-07	0.01	1	0.42	0.95	1.05	0.04
Lj0g3v0266719	hypothetical protein	-3.47	0	-2.76	2.62E-10	-2.33	0	2.91	3.66E-08	-0.44	0.32
Lj0g3v0202659	seed maturation protein PM39	-3.95	0	-2.9	0	-2.99	0	2.27	1.75E-03	0.25	1
Lj3g3v2039900	uncharacterised protein	-4.61	0	-2.95	8.59E-09	-2.9	0	2.99	8.81E-03	1.09	1
Lj4g3v2575050	70 kDa peptidyl-prolyl isomerase-like	-2.27	1.34E-03	-2.98	1.00E-06	-0.02	1	-0.76	0.85	-0.22	1
Lj0g3v0180709	uncharacterised protein	-3.77	0	-2.99	1.26E-09	-3.51	0	1.8	0.17	0.35	1
Lj0g3v0231189	uncharacterised protein	-5.02	6.43E-03	-3.2	3.56E-03	-5.27	5.25E-03	3.56	0.8	-2.69	0.97
Lj3g3v2809320	hypothetical protein	-5.47	2.11E-03	-4.83	3.45E-04	-4.04	2.70E-04	0.96	0.93	-0.57	1
Lj1g3v4202340	enolase-phosphatase E1-like	-7.45	2.05E-04	-5.93	2.77E-04	-2.34	0.83	-0.21	1	6.88	0.17
Lj4g3v0388280	mediator of RNA polymerase II transcription subunit	-2.64	8.52E-03	-9.36	2.95E-03	-9.43	4.20E-03	-0.56	0.51	-0.21	0.97

Values represent log2 fold change compared to H_2O controls
