# Supplementary material for: Distinct Lotus japonicus Transcriptomic Responses to a Spectrum of Bacteria Ranging From Symbiotic to Pathogenic
Source: Front Plant Sci. 2018 Aug 20;9:1218. doi: 10.3389/fpls.2018.01218 (PMC6110179; doi:10.3389/fpls.2018.01218)
Supplement: Supplementary file 4 [file Table_3.PDF]

Supplemental Table 3. Ps DC3000 differentially regulated genes

**Name**	**Gene annotation**	**Pst DC3000**	**FDR p-value**	**Ml R7A**	**FDR p-value**	**Be USDA61**	**FDR p-value**	**Rs JS763**	**FDR p-value**	**Sf HH103**	**FDR p-value**
**Lj1g3v2392240**	exportin-7-B-like, partial	10.67	0.01	11.1	1.41E-03	11.13	1.24E-03	11.37	4.44E-04	8.46	0.06
Ljchlorog3v0015200	hypothetical protein	10.52	0.03	0	0	0	0	12.76	1.44E-04	10.24	4.16E-03
Lj3g3v2330900	U-box domain-containing protein 33-like	5.96	0.04	4.19	0.61	2.9	1	5.19	0.04	2.73	1
Lj1g3v4669290	Thaumatin-like protein	5.77	4.04E-05	4.53	0.1	0.75	1	6.84	2.13E-07	-3.43	1
Lj6g3v0001230	calmodulin-like protein 11-like	4.91	0.05	3.99	0.29	-0.91	1	-3.39	0.59	-5.43	1
Lj4g3v2882250	bidirectional sugar transporter SWEET15-like	4.18	5.44E-03	2.27	1	2.85	0.72	-0.71	1	3.49	0.05
**Lj6g3v2170740**	pathogenesis-related protein 1a	4.07	3.68E-07	7.12	5.28E-08	-0.26	1	6.48	2.12E-12	0.96	1
**Lj5g3v0101610**	GYF domain-containing protein	3.91	0.03	3.63	2.90E-03	3.28	5.64E-04	3.7	5.49E-04	2.64	0.05
**Lj0g3v0095839**	receptor-like protein 12-like	3.88	0.03	3.74	0.04	4.13	0.01	3.7	0.03	2.91	0.7
Lj4g3v1684340	ZPT4-4	3.64	4.09E-03	2	1	2.5	1	2.38	0.54	2.23	1
Lj1g3v4549020	ankyrin repeat-containing protein	3.49	0.03	2.17	1	2.51	1	3.19	0.19	3.57	2.81E-03
Lj2g3v2002950	anthocyanidin synthase	3.41	0.01	0.88	1	0.13	1	-0.5	1	0.63	1
Lj0g3v0276859	glutathione S-transferase F11-like	3.4	0.01	0.14	1	1.18	1	2.47	0.2	3.45	5.93E-03
Lj0g3v0115609	MATE efflux family protein DTX1-like	3.25	0.02	1.16	1	-0.8	1	0.75	1	1.39	1
Lj6g3v1201340	R2R3-MYB transcription factor LjTT2a	2.63	1.06E-03	-0.74	1	-0.54	1	-1.37	0.36	0.24	1
Lj2g3v1370290	hypothetical protein	2.6	9.40E-03	0.82	1	1.22	1	0.71	1	0.55	1
**Lj0g3v0331049**	probable leucine-rich repeat receptor-like protein kinase At1g35710-like	2.44	9.75E-05	2.24	7.18E-04	2.62	1.28E-04	1.99	0.06	2.45	1.00E-03
Lj5g3v1414150	MtN26 protein	2.4	0.04	1.61	0.35	1.16	1	3.3	9.42E-04	1.26	1
Lj0g3v0164569	hypothetical protein	2.33	0.01	0.12	1	0.32	1	-0.39	1	0.66	1
Lj5g3v1988770	NADPH oxidase	2.31	0.05	0.62	1	1.69	0.38	0.88	1	0.19	1
Lj2g3v0914760	lupeol synthase	2.24	0.01	0.18	1	0.32	1	-0.41	1	0.63	1
Lj2g3v0914630	lupeol synthase	2.23	0.01	0.22	1	0.39	1	-0.25	1	0.55	1
**Lj5g3v1961260**	chitinase	2.23	1.54E-04	2.72	0.01	0.96	1	5.2	0	1.14	0.39
Lj6g3v0325210	lipid transfer protein precursor	2	6.36E-06	1.26	0.16	1.09	0.6	2.89	1.15E-06	0.53	1
Lj3g3v3069020	membrane transporter	-2.01	6.69E-07	-0.86	0.51	-1.23	0.36	-0.3	1	-1.56	1.71E-04
Lj0g3v0112969	uncharacterised protein	-2.09	1.75E-03	-0.77	1	-1.21	1	-1.16	0.67	-6.32E-03	1
Lj0g3v0331569	actin-like protein	-2.12	0.02	-0.61	1	-1.64	0.32	-0.05	1	-0.88	1
**Lj6g3v2274900**	ribosomal protein L27	-2.16	7.88E-03	-2.16	1.33E-04	-0.76	1	-1.14	0.5	-1.88	1.37E-03
Lj0g3v0364049	isoliquiritigenin 2'-O-methyltransferase-like	-2.24	1.18E-03	3.65E-03	1	-2.52	4.66E-06	1.04	0.51	-0.38	1
Lj3g3v3069010	membrane transporter	-2.25	2.01E-05	-0.69	1	-1.28	1	0.04	1	-1.08	0.15
Lj1g3v3904290	light-regulated protein-like	-2.36	2.12E-04	-0.12	1	-0.95	1	-0.12	1	-0.06	1
Lj0g3v0326379	uncharacterised protein	-2.41	0.04	0.05	1	-0.31	1	1.17	0.49	-0.16	1
Lj0g3v0090109	hypothetical protein	-2.65	6.69E-07	-0.33	1	-2.01	0.02	1.12	0.35	-1.23	0.67
Lj4g3v0451330	uncharacterised protein	-2.76	1.18E-03	0.94	1	-1.19	1	1.82	0.05	-0.25	1
Lj0g3v0322659	pentatricopeptide repeat-containing protein	-3.19	0.02	-2.1	0.29	-2.67	0.14	-0.48	1	-1.37	1
Lj1g3v0130160	WEB family protein	-3.32	1.11E-09	-2.09	0.27	-2.68	0.03	-1.44	0.64	-0.43	1
Lj0g3v0069389	cytochrome P450 82A3-like	-3.38	0.04	-1.01	1	-1.23	1	-0.81	1	-0.39	1
Lj4g3v0451200	CASP-like protein	-3.64	0.01	0.5	1	0.14	1	-1.63	0.53	0.3	1
Lj3g3v3069030	membrane transporter	-3.67	2.92E-05	-1.77	1	-0.05	1	-3.6	1.21E-03	-4.2	4.22E-05
Lj6g3v1018510	tRNA (guanine(37)-N1)-methyltransferase 2-like	-5.54	0.02	-3.34	0.3	-7.98	0.4	-1.07	1	-2.82	0.59
Lj5g3v1235710	alpha-galactosidase-like	-6.48	7.31E-07	-1.25	1	-2.92	0.67	-2.15	0.48	-2.63	0.54
Lj0g3v0099309	chromophore lyase CpcT/CpeT	-7.14	6.60E-04	0.14	1	-3.71	0.25	1.14	1	-6.44	2.63E-04
Lj0g3v0300529	protein phosphatase 1 catalitic subunit	-7.25	1.27E-05	-2.43	1	0.28	1	0.07	1	-0.95	1
**Lj0g3v0103099**	agenet domain-containing protein / BAH domain-containing protein	-10	0.02	-9.88	0.02	-2.58	0.16	-9.8	0.01	-2.18	0.95

Values represent log2 fold change compared to H2O controls

Genes in bold represent those similarly differntially regulated between Pst DC3000 and Ml R7A
