# Supplementary material for: Distinct Lotus japonicus Transcriptomic Responses to a Spectrum of Bacteria Ranging From Symbiotic to Pathogenic
Source: Front Plant Sci. 2018 Aug 20;9:1218. doi: 10.3389/fpls.2018.01218 (PMC6110179; doi:10.3389/fpls.2018.01218)
Supplement: Supplementary file 5 [file Table_4.PDF]

Supplemental Table 4. Ml R7A differentially expressed genes not similarly regulated by incompatible rhizobia

Name	Gene annotation	Ml R7A	FDR p-value	Sf HH103	FDR p-value	Be USDA61	FDR p-value
Lj4g3v1983610	Full=Early nodulin-16;	11.74	7.50E-04	6.82	0.38	NaN	NaN
Lj0g3v0010159	WD-repeat protein	11.13	1.59E-03	6.47	0.44	1.99	1
Lj3g3v1855560	auxin-induced protein 5NG4-like	9.82	0.02	6.28	0.59	1.99	1
Lj1g3v0415090	probable inactive receptor kinase At2g26730-like	9.61	0.02	7.9	0.11	1.99	1
Lj4g3v2618540	multifunctional transport intrinsic membrane protein 2	9.09	0.04	7.54	0.15	1.99	1
Lj5g3v1511500	topless-related protein 3-like	8.98	0.04	5.6	1	2.8	1
Lj6g3v2170740	pathogenesis-related protein 1a	7.12	5.28E-08	0.96	1	-0.26	1
Lj4g3v2365210	nuclear transcription factor Y subunit B-5-like	6.09	7.65E-03	3.11	1	0.22	1
Lj0g3v0000559	CLAVATA3/ESR (CLE)-related protein 6-like	5.59	0.04	2.82	1	0.22	1
Lj1g3v5021140	subtilisin-like protease-like	4.49	3.66E-03	3.05	0.36	0.27	1
Lj0g3v0062279	hypothetical protein	4.41	0.04	-3.39	1	0.36	1
Lj0g3v0171159	extensin-1-like isoform X1	3.93	0.01	0.51	1	-5.91	1
Lj2g3v0743220	leucine-rich repeat receptor-like protein	3.92	0.02	-4.82	1	-1.99	1
Lj2g3v1014120	MtN24	3.66	4.63E-03	2.27	0.73	-4.36	1
Lj3g3v0819890	hypothetical protein	3.38	0.04	2.81	0.39	-4.37	1
Lj3g3v0323320	calmodulin-binding family protein isoform 1	3.28	0.02	0.73	1	0.32	1
Lj0g3v0188899	cytochrome P450	3.18	0.04	2.99	0.17	0.96	1
Lj1g3v0416320	formin-like protein 8-like	2.96	0.01	1.21	1	1.13	1
Lj1g3v2418690	Wound-induced protein	2.94	7.05E-03	2.08	0.09	0.66	1
Lj5g3v2288900	chalcone isomerase	2.89	0.02	1.15	1	0.7	1
Lj5g3v1961260	chitinase	2.72	0.01	1.14	0.39	0.96	1
Lj0g3v0146729	probable WRKY transcription factor 72-like	2.6	1.64E-03	1.84	0.25	0.66	1
Lj6g3v0933470	4-coumarate--CoA ligase 2-like isoform X1	2.19	9.72E-03	-7.36E-04	1	2.22	0.63
Lj6g3v0098610	DNA ligase 1-like	2.18	4.64E-03	1.57	0.16	1.91	0.04
Lj5g3v2027290	multidrug and toxin extrusion protein 1-like	2.13	6.44E-09	1.69	2.11E-06	-2.68E-04	1
Lj5g3v1174420	spc97 / Spc98 family of spindle pole body (SBP) component	2.04	9.16E-07	1.36	9.83E-03	0.2	1
Lj6g3v2274900	ribosomal protein L27 homolog	-2.16	1.33E-04	-1.88	1.37E-03	-0.76	1
Lj3g3v2809600	probable L-type lectin-domain containing receptor kinase	-2.17	8.76E-03	-0.34	1	-0.44	1
Lj0g3v0012529	cationic peroxidase 1-like	-2.44	9.95E-03	-1.24	1	-1.65	0.58
Lj1g3v3975520	Anthranilate N-benzoyltransferase protein	-2.6	0.04	-0.02	1	-2.95	0.08
Lj0g3v0078579	pentatricopeptide repeat-containing protein At3g22690-like	-3.48	0.02	-0.59	1	-1.73	1
Lj3g3v0838370	cellulose synthase A catalytic subunit 3	-3.58	0.04	-0.88	1	-8.16	0.31
Lj6g3v0184200	Kinesin-like protein	-4.8	2.44E-05	0.17	1	-0.85	1
Lj0g3v0103099	Bromo-adjacent homology (BAH) domain-containing protein	-9.88	0.02	-2.18	0.95	-2.58	0.16

Values represent log2 fold change compared to H_2O controls
