# Supplementary material for: Distinct Lotus japonicus Transcriptomic Responses to a Spectrum of Bacteria Ranging From Symbiotic to Pathogenic
Source: Front Plant Sci. 2018 Aug 20;9:1218. doi: 10.3389/fpls.2018.01218 (PMC6110179; doi:10.3389/fpls.2018.01218)
Supplement: Supplementary file 6 [file Table_5.PDF]

Supplemental Table 5. Rs JS763 induced gene expression overlapping with the early symbiosis differential gene expression set

Name	Gene annotation	Rs JS763	FDR p-value	Root_R7A_3dpi	FDR p-value	RH_R7A_3dpi	FDR p-value	RH_NF_2dpi	FDR p-value
Lj6g3v0574840	uncharacterised protein	10.36	5.80E-03	1.19	0.92	3.86	6.14E-04	2.49	0.43
Lj3g3v2027230	beta-amyrin synthase	6.75	7.37E-04	2.71	0.36	3.58	2.73E-05	7.62	0.08
Lj4g3v2253780	serpin-ZX-like	4.56	0.01	2.61	0	7.71	0	4.87	0
Lj0g3v0258029	protein kinase-related protein precursor	4.27	8.98E-04	0.12	1	0.46	0.62	9.12	0.03
Lj0g3v0054209	ribosomal protein S4	3.83	4.05E-06	2.4	2.00E-03	-1.12	0.96	1.67	1
Lj0g3v0270389	uncharacterised protein	3.48	0.02	3.02	3.13E-04	0.7	0.77	2.92	0.88
Lj4g3v1855730	early nodulin 93-like protein	3.39	1.31E-03	2.99	0	2.15	0.58	0.94	0.93
Lj5g3v1414150	MtN26 protein	3.3	9.42E-04	2.84	0	1.57	0	1.12	4.06E-09
Lj0g3v0285809	hypothetical protein	3.24	1.49E-10	2.08	3.84E-03	-0.45	0.25	-0.57	0.03
Lj0g3v0286359	disease resistance response protein, PR10-like	3.03	8.00E-03	2.84	0.13	-0.21	1	3.07	7.41E-05
Lj0g3v0244249	hypothetical protein	2.9	4.12E-06	2.19	8.42E-05	0.02	1	-0.23	0.92
Lj1g3v3975870	uncharacterised protein	2.9	8.00E-03	2.86	5.60E-03	1.25	0.22	0.91	0.81
Lj0g3v0192559	hypothetical protein	2.77	1.92E-05	2.81	1.08E-03	-0.23	0.67	-0.19	1
Lj0g3v0050699	maturase K	2.62	2.53E-04	2.21	7.46E-06	0.08	1	-0.29	1
Lj6g3v1946360	uncharacterised protein	2.6	0.03	1.96	1.23E-12	0.25	0.97	2.4	1.73E-05
Ljchlorog3v0000230	hypothetical protein	2.59	9.03E-08	2.26	4.39E-03	0.06	1	0.15	1
Lj6g3v0509430	salicylate O-methyltransferase-like	2.54	9.26E-04	5.91	7.34E-11	4.07	0	1.17	0.54
Ljchlorog3v0000040	ribulose-1,5-bisphosphate carboxylase/oxygenase large subunit	2.48	9.19E-06	2.22	5.97E-05	-0.06	1	-0.16	1
Lj0g3v0124339	ATPase subunit 1 (mitochondrion)	2.47	1.68E-06	2.33	1.40E-06	-0.13	0.86	-0.36	0.74
Ljchlorog3v0010940	Ljchlorog3v0010940	2.46	1.15E-06	2.13	4.46E-04	0.05	1	-0.49	0.86
Lj2g3v0776860	sst1 protein	2.46	0.01	1.19	0	2.58	0	2.35	3.90E-04
Ljmitog3v0000660	Cytochrome c oxidase subunit 3	2.46	6.14E-07	2.09	9.39E-06	-0.14	0.75	-0.16	0.95
Lj0g3v0053799	ycf2	2.44	9.23E-03	2.49	5.48E-04	-0.18	1	1.26	0.96
Lj0g3v0054319	ribulose-1,5-bisphosphate carboxylase/oxygenase large subunit	2.44	1.53E-06	2.03	8.27E-03	-0.04	1	0.06	1
Lj0g3v0225279	hypothetical protein	2.43	1.86E-04	2.57	3.68E-05	-0.06	1	-0.44	0.32
Lj5g3v0692300	cytokinin dehydrogenase 3-like	2.43	2.94E-03	0.79	1.82E-03	2.21	1.55E-09	-0.75	0.28
Ljchlorog3v0016130	Ljchlorog3v0016130	2.42	3.01E-06	2.03	1.21E-03	0.04	1	-0.65	0.65
Lj1g3v4093460	hypothetical protein	2.41	1.14E-04	2.2	3.16E-04	-6.52E-03	1	-0.39	0.37
Lj0g3v0054259	NADH dehydrogenase subunit J	2.39	0.02	2.59	3.10E-03	-0.58	0.96	1.67	1
Lj4g3v0149070	hypothetical protein	2.37	2.42E-04	2.57	3.04E-05	-0.06	0.99	-0.39	0.49
Lj4g3v0148990	hypothetical protein	2.35	2.42E-04	2.57	2.34E-05	-0.06	1	-0.43	0.46
Lj2g3v0045000	hypothetical protein	2.33	4.91E-06	2.25	7.80E-05	-0.2	0.41	-0.42	0.36
Lj4g3v0136680	hypothetical protein	2.33	2.12E-04	2.43	5.43E-05	-0.09	0.95	-0.44	0.33
Lj4g3v1332890	hypothetical protein	2.33	8.92E-06	2.32	8.38E-05	-0.12	0.87	-0.14	1
Lj0g3v0027139	hypothetical protein	2.32	2.84E-04	2.54	7.16E-05	-0.25	0.14	-0.56	0.63
Lj4g3v0149200	hypothetical protein	2.32	2.62E-04	2.48	1.03E-04	-0.18	0.46	-0.53	0.67
Lj4g3v3015210	phospholipase A2-alpha-like	2.32	0.02	2.15	0.05	-0.37	0.52	-0.8	0.66
Lj0g3v0002619	uncharacterised protein	2.31	2.55E-04	2.57	2.97E-05	-0.03	1	-0.44	0.44
Lj0g3v0054159	photosystem I P700 chlorophyll a apoprotein A2	2.28	3.05E-04	2.12	1.54E-04	-0.54	0.3	-0.22	1
Ljmitog3v0000500	hypothetical protein	2.28	3.64E-04	2.34	6.58E-05	-0.09	0.93	-0.38	0.68
Lj4g3v0214620	hypothetical protein	2.26	0.03	2.75	1.54E-03	-0.35	0.02	-0.51	0.76
Lj2g3v1645520	phosphoserine aminotransferase, chloroplastic-like	2.25	5.98E-05	-0.14	1	3.04	0.03	1.67	1
Lj3g3v2054320	cytochrome oxidase subunit III	2.25	2.48E-03	2.04	6.43E-04	0.31	0.71	0.22	1
Ljmitog3v0000310	hypothetical protein	2.23	0.03	2.42	0.01	-0.18	0.97	-0.79	0.75
Lj0g3v0218689	hypothetical protein	2.22	4.20E-04	2.46	9.81E-05	-0.17	0.56	-0.51	0.71
Lj0g3v0195869	hypothetical protein	2.19	1.22E-05	2.23	8.86E-05	1.11	0.22	-1.65	0.18
Lj4g3v1332860	uncharacterised protein	2.18	4.98E-05	2.14	7.21E-04	-0.17	0.78	-0.36	0.93
Lj1g3v2776600	peptidase M24	2.17	4.09E-04	2.34	1.67E-04	-0.16	0.61	-0.46	0.78
Lj0g3v0199449	ribosomal protein S4, mitochondrial-like	2.16	1.31E-03	2	1.41E-06	-0.17	0.88	0.35	1
Lj0g3v0244459	hydroquinone glucosyltransferase	2.16	0.03	8.11	0.04	6.28	1.30E-05	2.27	0.65
Lj0g3v0104129	photosystem Q(B) protein	2.13	4.39E-04	2.35	4.42E-03	-0.25	0.99	0.96	1
Ljchlorog3v0010790	cytochrome b6/f complex subunit IV (chloroplast)	2.08	2.61E-03	2.1	5.95E-04	0.43	0.96	-1.85	0.55
Lj0g3v0054059	photosystem II protein D2	2.07	9.83E-05	2.02	8.38E-08	-0.41	0.4	0.5	0.89
Lj0g3v0112839	bowman-Birk type protease inhibitor-like precursor	2.07	1.42E-06	0.74	0.03	2.17	1.10E-03	0.38	1
Ljchlorog3v0015040	hypothetical protein	2.06	7.72E-05	2.19	9.44E-04	-0.04	1	-0.19	1
Lj0g3v0247849	hypothetical protein	2.01	2.50E-04	2.18	2.11E-03	-0.06	1	-0.19	1
Lj1g3v0913340	WAT1-related protein At1g70260-like	-2.43	4.95E-03	-1.29	0	-4.49	0	0.81	5.63E-05
Lj5g3v1501480	probable protein phosphatase 2C 73-like	-2.93	0.05	-0.13	1	NaN	NaN	-2.31	0.04
Lj3g3v3069030	membrane transporter	-3.6	1.21E-03	-2.44	0	-5.7	0	0.66	0.12
Lj5g3v0112000	phosphatidylinositol/phosphatidylcholine transfer protein	-6.47	1.66E-03	-8	0.03	-0.41	0.72	-0.26	1

Values represent log2 fold change compared to H_2O controls
