# Supplementary material for: Distinct Lotus japonicus Transcriptomic Responses to a Spectrum of Bacteria Ranging From Symbiotic to Pathogenic
Source: Front Plant Sci. 2018 Aug 20;9:1218. doi: 10.3389/fpls.2018.01218 (PMC6110179; doi:10.3389/fpls.2018.01218)
Supplement: Supplementary file 7 [file Data_Sheet_1.DOCX]

##**Example MDS script**##

﻿##From fig 3d, spectrum of interacting bacteria##

##Requires combinat and Possionseq packages##

d <- read.delim("20171009_Terry_symdef_for_R.txt")

colnames(d)

#remove bad reps

d <- d[,c(-2,-6,-11,-15,-18,-22,-26,-27,-28,-29)]

colnames(d)

## Normalize using PoissonSeq

library(PoissonSeq)

norm.facs.pss <- PS.Est.Depth(d[,2:ncol(d)], iter=5, ct.sum=5, ct.mean=0.2)

norm.facs.pss

d.norm.pss <- t(t(d[,2:ncol(d)])/norm.facs.pss)

d <- cbind(as.data.frame(d[,1]), d.norm.pss)

colnames(d)[1] <- "Name"

head(d)

# make function to compute row variance

RowVar <- function(x) {

  rowSums((x - rowMeans(x))^2)/(dim(x)[2] - 1)

}

# For each gene, calculate variance across all samples

# and across replicates for each sample

d$tot.var <- RowVar(d[,2:19]) # all samples

d$sam1.var <- RowVar(d[,2:4]) # Gifu mock

d$sam2.var <- RowVar(d[,5:7]) # Sf

d$sam3.var <- RowVar(d[,8:10]) # M loti

d$sam4.var <- RowVar(d[,11:13]) # Be

d$sam5.var <- RowVar(d[,14:16]) #pst

d$sam6.var <- RowVar(d[,17:19]) # RsJs

# filter genes for total variance and read counts

nrow(d)

hist(log(d$tot.var,10), breaks=50)

d.filt <- subset(d, tot.var > 10)

nrow(d.filt)

# filter for total read counts

hist(log10(rowSums(d.filt[,2:19])))

d.filt <- subset(d.filt, rowSums(d.filt[,2:19])> 100)

nrow(d.filt)

# For each gene, generate random groups of "replicates"

# and calculate variance for comparison with the observed replicate variance

k=100     #number of iterations

rep.num =3     #number of replicates

i=1

for (i in 1:k) {

ran.cols <- sample(ncol(d.filt[,2:19]), rep.num) # sample random columns

ran.cols <- ran.cols + 1 # adjust index

colnum <- ncol(d.filt) + 1 # specify index of column to be added

d.filt[,colnum] <- RowVar(d.filt[,ran.cols]) #add random replicate variance

}

head(d.filt)

summary(d.filt)

# calculate mean of observed replicate variances

d.filt$mean.rep.var <- (d.filt$sam1.var + d.filt$sam2.var + d.filt$sam3.var + d.filt$sam4.var + d.filt$sam5.var + d.filt$sam6.var)/6

# calculate mean of random replicate variances

d.filt$mean.ran.var <- rowMeans(d.filt[,27:126])

# Add ratio between observed replicate and random replicate variances

d.filt$ratio.var <- log(d.filt$mean.rep.var/d.filt$mean.ran.var,2)

hist(d.filt$ratio.var, breaks=100)

# filter based on the ratios

d.filt.sorted <- d.filt[order(d.filt$ratio.var),]

d.filt.sorted <- subset(d.filt.sorted, ratio.var > -Inf)

head(d.filt.sorted[,c(2:20,127:129)], 20)

tail(d.filt.sorted[,c(2:20,127:129)], 20)

hist(d.filt.sorted$ratio.var)

d.for.pca <- subset(d.filt.sorted, ratio.var < -1)

nrow(d.for.pca)

## PCA analysis

d.for.pca <- d.for.pca[,2:19]

d.for.pca <- log(1+d.for.pca)

#rownames(d.for.pca) <- d.sorted$Name

head(d.for.pca)

d.pca <- prcomp(d.for.pca, scale=TRUE)

par(mfrow=c(1,1))

plot(d.pca$rotation[,1],d.pca$rotation[,2], type="n")

text(d.pca$rotation[,1],d.pca$rotation[,2], rownames(d.pca$rotation), cex = 0.4)

heatmap(as.matrix(d.for.pca), cexCol=0.5)

# Classical MDS

# N rows (objects) x p columns (variables)

# each row identified by a unique row name

d <- dist(t(d.for.pca)) # euclidean distances between the rows

fit <- cmdscale(d,eig=TRUE, k=2) # k is the number of dim

fit # view results

# plot solution

x <- fit$points[,1]

y <- fit$points[,2]

plot(x, y, xlab="Coordinate 1", ylab="Coordinate 2",

  main="Metric MDS", type="n")

text(x, y, labels = colnames(d.for.pca), cex=.5)

##**Example Heatmap script**##

##From Fig 5c, Rs JS763 DGE compared to other samples##

##Requires heatmap3 package##

d <- read.table(file="fig4cv2.txt",header=FALSE)

rnames <- d[,1]

mat_d <- data.matrix(d[,2:12])

rownames (mat_d) <- rnames

colnames(mat_d) <- c("Ml_R7A","Sf_HH103","Be_USDA61","Ps_DC3000","Rs_Js763","Root_R7A_3dpi","Shoot_R7A_3dpi","RH_R7A_1dpi","RH_R7A_3dpi","RH_NF_2dpi","RH_nodC_1dpi")

heatmap3(as.matrix(mat_d), cexRow=0.25, cexCol=0.5, scale="row", Colv=NA, Rowv=NA)
